# Characterizing the effect of demographics, cardiorespiratory factors, and inter-subject variation on maternal heart rate variability in pregnancy with statistical modeling: a retrospective observational analysis

**DOI:** 10.1038/s41598-022-21792-2

**Published:** 2022-11-11

**Authors:** M. Bester, R. Joshi, A. Linders, M. Mischi, J. O. E. H. van Laar, R. Vullings

**Affiliations:** 1 Department of Electrical Engineering, Eindhoven University of Technology, 5612 AZ Eindhoven, The Netherlands; 2 Patient Care and Monitoring, Philips Research, 5656 AE Eindhoven, The Netherlands; 3 Faculty of Health, Medicine and Life Science, Maastricht University, 6200 MD Maastricht, The Netherlands; 4 Department of Obstetrics and Gynecology, Máxima Medical Centrum, De Run 4600, 5504 DB Veldhoven, The Netherlands

**Keywords:** Autonomic nervous system, Biomedical engineering, Statistical methods

## Abstract

Pregnancy complications are associated with insufficient adaptation of the maternal autonomic nervous system to the physiological demands of pregnancy. Consequently, assessing maternal heart rate variability (mHRV)—which reflects autonomic regulation—is a promising tool for detecting early deterioration in maternal health. However, before mHRV can be used to screen for complications, an understanding of the factors influencing mHRV during healthy pregnancy is needed. In this retrospective observational study, we develop regression models to unravel the effects of maternal demographics (age, body mass index (BMI), gestational age (GA), and parity), cardiorespiratory factors (heart rate and breathing rate), and inter-subject variation on mHRV. We develop these models using two datasets which are comprised of, respectively, single measurements in 290 healthy pregnant women and repeated measurements (median = 8) in 29 women with healthy pregnancies. Our most consequential finding is that between one-third and two-thirds of the variation in mHRV can be attributed to inter-subject variability. Additionally, median heart rate dominantly affects mHRV (*p* < 0.001), while BMI and parity have no effect. Moreover, we found that median breathing rate, age, and GA all impact mHRV (*p* < 0.05). These results suggest that personalized, long-term monitoring would be necessary for using mHRV for obstetric screening.

## Introduction

Assessing heart rate variability (HRV) offers a non-invasive opportunity for monitoring autonomic activity^1^. HRV has been used to assess cardiac health, predict short-term mortality in emergency-room patients, investigate fetal well-being^2^, and—through longitudinal and continuous monitoring—detect conditions such as sepsis and Covid-19 infection before the onset of observable symptoms^1,3–5^. More recently, investigations have focused on the association between the HRV of the mother during pregnancy—henceforth referred to as maternal HRV (mHRV)—and maternal health, in large part driven by the need for tools for the early detection of pregnancy complications^6,7^. The inability to detect these complications early enough to implement risk-mitigating interventions remains a barrier to reducing perinatal mortality and morbidity^8^. For example, the increase in blood pressure symptomatic of pregnancy induced hypertension only arises after 20 weeks of gestation pregnancy, which is beyond the window in which the clinically available suite of interventions has an optimal impact^8,9^.

Motivated by the suspected autonomic dysfunction associated with preeclampsia (a type of hypertensive disorder in pregnancy), Eneroth et al. were amongst the first to investigate mHRV in complicated pregnancies^10^. Further investigations not only confirmed their initial result that preeclamptic women had altered mHRV in comparison to healthy pregnancies^9^ but also demonstrated similar findings in other pregnancy complications^11,12^. Consequently, assessing mHRV may offer a tool for identifying pregnancy complications before the onset of the typical symptoms associated with the complication^13,14^.

Despite the potential of HRV as an obstetric screening method, interpreting HRV is challenging due to the sensitivity of the metric to a multitude of factors^1,15^. For instance, HRV features have a well-documented relationship with cardiorespiratory factors^16,17^ and have also been shown to be influenced by demographics such as age and body mass index (BMI)^18–20^. However, apart from a single study analyzing only frequency domain features of HRV^21^, these associations have not been investigated in a pregnant population. Notably, pregnancy alters autonomic regulation, and these regulatory effects change through the course of advancing gestation^6,22^. Therefore, it is imperative to establish an understanding of how maternal demographics influence mHRV in a healthy pregnancy to, in turn, be able to identify abnormal values of mHRV.

In this paper, we describe the effects of maternal characteristics on selected mHRV features using regression modeling. In all cases, the null hypothesis being tested is that the maternal characteristic does not affect mHRV. We analyze two datasets to test this hypothesis. First, we develop a multiple linear regression model based on a dataset of single measurements in 290 healthy pregnant women to characterize the effects of maternal demographics and cardiorespiratory factors on mHRV. Second, we analyze a dataset of repeated measurements (median of eight per participant) taken over the course of 29 healthy pregnancies to develop a linear mixed-effects model. This model allows for discerning the inter-subject variability by making use of these repeated measurements. Finally, considering the results from both models, we discuss their implications for using mHRV as an obstetric screening tool.

## Methods

### Study design and population

#### Datasets

This study is a retrospective observational analysis of two existing datasets of abdominal ECG measurements (from which maternal R-peaks can be extracted). The first dataset, referred to as *Dataset 1*, contains abdominal ECG recordings (NEMO Healthcare BV, the Netherlands) from 494 women with singleton pregnancies between 18 and 24 weeks of gestation^23^. Measurements of approximately 30-min duration were acquired at 500 Hz while women were lying in a semi-recumbent position. The study was conducted between May 2014 and February 2017. The study protocol for the original study has been previously described^23^. For our analysis, women with missing information on BMI, age, and gestational age (GA) were excluded (n = 79). Furthermore, women with maternal pregnancy complications such as preeclampsia or gestational diabetes, health conditions such as asthma, hyperthyroidism, or heart disease, or who were taking any medications (e.g., anti-coagulants, anti-hypertensives, psychotropics) except vitamins were excluded (n = 121). Finally, women with more than 25% unreliable data in their recordings (as defined in the "Preprocessing" section) were excluded (n = 4), resulting in a total of 290 participants. Of the participants included in the analysis, 74 were diagnosed with fetal congenital heart disease (CHD). These participants were not excluded, since there is no evidence that fetal CHD would affect mHRV. However, this assumption is assessed during the model development (see section: "Statistical modeling"). Patient characteristics are presented in Table 1.

**Table 1:** Characteristics of Dataset 1.

Characteristic	Dataset 1
Number of included participants	290
Number of measurements	290
Age	30 (28–34) years
BMI before pregnancy	22.7 (20.7–25.9) kg/m^2^
Gestational age at measurement	20 weeks 3 days (19 weeks 4 days–21 weeks 3 days)
Nulliparous	54.5%
Fetal CHD	78 cases (26.9%)
Measurement length	30.8 (29.1–32.3) min

Where applicable, values are presented as median and interquartile range.

The second dataset, *Dataset 2*, was collected between 2008 and 2009. Healthy women (18 years and older) with uneventful, singleton pregnancies were recruited before 12 weeks of gestation for participation (n = 40)^24^. Abdominal ECG measurements (the NEMO device, Maastricht Instruments, the Netherlands) of approximately 45 min were obtained at a sampling rate of 1000 Hz. Recordings were done between 08:00 and 18:00 while women were lying comfortably in a semi-recumbent position. Repeated measurements were performed at approximately 14, 18, 22, 24, 26, 30, 34, 36, 38, and 40 weeks of gestation. The seven women who developed complications and the four for whom all ECG data were missing were subsequently excluded, resulting in a cohort of 29 women with a total of 248 ECG recordings. ECG recordings with more than 25% unreliable data (see: "Preprocessing") were also removed from the analysis, resulting in a total of 230 measurements and a median of eight measurements per participant (interquartile range: 7–9). Participants took no medication apart from iron supplements or vitamins. Table 2 outlines the characteristics of the included participants. The original study has been described previously^24^.

**Table 2:** Characteristics of Dataset 2.

Characteristic	Dataset 2
Number of included participants	29
Number of measurements	230
Age	31 (28–34) years
BMI	22.9 (20.9–26.1) kg/ m^2^
Gestational age at birth	39 weeks 6 days (38 weeks 6 days–41 weeks 3 days)
Nulliparous	65.5%
Fetal CHD	0%
Measurement length	44.8 (40.9–46.1) min

Where applicable, values are represented as median and interquartile ranges.

All participants provided written informed consent. The institutional review board at the Máxima Medical Center, Veldhoven, the Netherlands, approved the original studies (DS1: NL48535.015.14; DS2: reference number 0650), which were performed in accordance with the Declaration of Helsinki. The same review board granted a waiver for this secondary analysis in 2021 (reference number N21.008) in accordance with the Dutch law on medical research with humans.

### Signal processing and calculating HRV features

#### Preprocessing

Multichannel abdominal ECG measurements were filtered by applying a 4th order Butterworth bandpass filter of 1–70 Hz to suppress out-of-band noise and artifacts and a notch filter at 50 Hz to suppress powerline interference. Thereafter, a fixed linear combination of the various abdominal channels was applied to enhance maternal QRS peaks^25^ and R-peaks were detected with a previously reported algorithm^22,26^. These R-peaks were used to determine the corresponding tachograms (i.e., the sequence of durations of the RR intervals). RR intervals that were outside of a realistic physiological range (0.4–2 s) or differed from preceding RR intervals by more than 20% were rejected^27–29^. Furthermore, RR intervals for which both the preceding and following values were excluded based on the above criteria, were also excluded. For HRV features that require a continual time series, the missing values were linearly interpolated.

#### Cardiorespiratory factors

In our analysis, we aim to determine the effect of cardiorespiratory factors on mHRV. To this end, the median heart rate (HR) was calculated for each ECG measurement in beats per minute (bpm). Furthermore, the BR was estimated from the tachogram by applying empirical mode decomposition (EMD)^30,31^. EMD performs a time adaptive decomposition of a complex signal into elementary components that do not overlap in frequency. These extracted components have well-behaved Hilbert transforms from which the instantaneous frequencies can subsequently be determined. As respiration is the highest frequency oscillation contributing to HRV, the first decomposition is taken as the respiratory modulation^31^. The BR was calculated based on 2-min segments, moved along the total signal with a 50% overlap between segments. If more than 5% of the RR intervals in a segment were unreliable (as defined in the "Preprocessing" section), the entire segment was disregarded from the BR analysis. Information above 0.5 Hz and below 0.1 Hz was filtered out and the dominant remaining frequency was taken as the estimated BR per segment. The median of all the BRs calculated per measurement segment was taken as the median BR of the total measurement. The BR is presented in breaths per minute (brpm). For *Dataset 2*, which has multiple measurements per participant, no BR could be calculated for five measurements owing to a high occurrence of unreliable RR intervals. In these cases, the average median BR for that participant was used as a replacement. The median HR and BR per dataset are reported in Table 3.

**Table 3:** Cardiorespiratory and heart rate variability parameters, reported as median with interquartile range.

Variable	Dataset 1	Dataset 2
SDNN (ms)	54.0 (42.2–66.8)	47.2 (36.6–59.9)
RMSSD (ms)	29.6 (21.2–42.9)	22.5 (14.9–33.3)
SampEn (a.u.)	1.4 (1.2–1.6)	1.2 (0.9–1.4)
HR (bpm)	78.1 (71.5–84.0)	79.9 (72.6–87.3)
BR (brpm)	14.1 (13.2–15.2)	13.8 (12.8–15.1)

The processing of maternal RR intervals from fetal ECG measurements, as well as the development of statistical models described in the next section, was done in MATLAB (MathWorks, USA). All other processing was done in Python (PSF, USA).

*ms* milliseconds, *a.u.* arbitrary units, *bpm* beats per minute, *brpm* breaths per minute.

#### HRV features

Three HRV features were used for the analysis: SDNN (standard deviation of all NN intervals), RMSSD (root mean squared successive differences of NN intervals), and SampEn (sample entropy of HR)^15,32^. SDNN and RMSSD are the most widely used time-domain features for HRV^1^. SDNN reflects overall variability and is influenced by both sympathetic and vagal activity. RMSSD captures immediate beat-to-beat variability. Consequently, this feature mainly indexes vagal activity, which can influence immediate subsequent heartbeats^15,33^. Lastly, SampEn characterizes the complexity of the HR time series, with lower SampEn indicating a more regular signal^15^. In previous work, we also found that SampEn is particularly sensitive to healthily progressing gestation^22^. The medians of the HRV features per dataset are reported in Table 3.

### Statistical modeling and testing

Multiple linear regression models (MLRs) enable the quantification of the influence of multiple independent variables (IVs)—in our case, participant demographics and cardiorespiratory factors—on each of the three dependent variables (DVs), i.e., the HRV features. These models only incorporate fixed effects (FEs), which represent the effects of the IVs, i.e., age, BMI, GA, parity, median HR, and median BR. We developed MLRs for both datasets. Parity is considered a categorical variable, with participants being labeled either nulliparous or parous. Fetal CHD was also added as a categorical IV to test the assumption that this fetal condition does not affect mHRV. In all cases, the null hypothesis is that the maternal characteristics being investigated do not affect mHRV.

We assessed the fit of our models by performing the *F*-test. If the *F*-test of overall significance is statistically significant (*p* < 0.05), it indicates that the fit of the model with the FEs is significantly better than that of an intercept-only model (i.e., a model with only a constant term and no IVs). We also quantified the goodness-of-fit of our models by calculating the adjusted *R*^2^, i.e., the coefficient of determination. The adjusted *R*^2^ specifies what percentage of the variation observed in the DV can be explained by the model. For example, an adjusted *R*^2^ of 0.70 means that 70% of the variation in the DV can be attributed to the IVs in the model. The remaining 30% would then be a result of variables that were not incorporated into the model.

Another likely source of variances in the DVs is the possible inherent differences between subjects’ baseline mHRV. When multiple measurements are available per participant (as is the case in *Dataset 2*, where repeated measurements were recorded at different GAs throughout pregnancy), a linear mixed-effects model (LMM) can be developed to quantify this inter-subject variability. LMMs capture the influence of both FEs and random effects (REs). In our case, the REs correspond to an individual intercept which is estimated for each participant (as opposed to the single intercept estimated in the MLRs). Subsequently, we developed an LMM for *Dataset 2*. We compared the LMM against an FE-only model with the log-likelihood ratio test to test whether adding the REs significantly improves the fit. Finally, the intra-class correlation (ICC), which is the ratio of the variance of the random intercept to the total variance, was calculated to determine how much of the overall variance in DVs can be explained by inter-subject variability^33^.

### Model development and diagnostics

Before developing the model, multicollinearity between the IVs was assessed by calculating their variable inflation factors (VIF). A VIF of between one and five is acceptably low. All VIFs were between one and two; subsequently, all IVs were included in the model. Furthermore, the distributions of the DVs were checked, since LMMs are typically more appropriate for normally distributed DVs. SDNN and RMSSD were right-skewed and subsequently log-transformed to yield a more normal distribution. SampEn, which was originally left-skewed, was more normally distributed once the values were squared. Hereafter, MLRs were developed for both datasets and LMMs were developed for *Dataset 2* (which included repeated measurements per participant). The models were developed for each of the three DVs in the datasets.

Several checks were implemented after the models had been developed to assess their validity and to check whether the appropriate statistical assumptions were satisfactorily met. The following plots were generated to check these assumptions^33^:

1. Normal probability plots of the residuals of the models (i.e., the error between the predicted value and the observed value) to visually assess whether the residuals were normally distributed.
2. Plots of fitted values versus the residuals to identify heteroscedasticity.
3. Plots of IVs and residuals to determine whether there are trends in the data that suggest that it would be appropriate to transform IVs before modeling them.
4. Plots of residuals versus leverage with overlaid contour plots of Cook’s distance to identify and characterize the effect of any outliers. Leverage measures the distance between an observation and the mean value of the remaining observations; in essence, it measures the unusualness of the observation. Cook’s distance is a measure of the influence of an observation in changing the slope of the regression line.
5. The histogram of the random effect to identify whether the random intercept was roughly normally distributed and that no individual subject exhibits patterns distinctly different from the rest.

### Model interpretation

The *F*-statistic and its corresponding *p*, along with the adjusted *R*^2^ are reported for each model. Furthermore, the ICC is reported for the LMMs. Since all the DVs were transformed before modeling, the MLRs and LMMs, in effect, modeled DVs which have non-linear relationships with the IVs. Therefore, instead of reporting the regression table, we plot the effects of all IVs (with 95% confidence intervals, CI) against the suitably transformed DVs for ease of interpretation. Where the effect of an IV is significant, the corresponding *p* is reported on the plot. For these plots, the IVs were varied between the 5th to 95th percentile ranges of values, as estimated from the corresponding dataset, while all other independent variables were held constant at their corresponding median levels.

## Results

Statistical models were independently developed on both datasets to explain the variation observed in three mHRV features: SDNN, RMSSD, and SampEn. All models developed were significantly better (*p* < 0.001) at explaining this variation than a model consisting of only a constant term. Concerning *Dataset 1*, fetal CHD was initially added as a categorical IV but had no significant or discernable effect on the DVs. Subsequently, fetal CHD was removed as an IV and no further distinction was made between participants with fetal CHD and those without. For the models based on *Dataset 2*, adding REs to the FEs significantly improved the model for all DVs (*p* < 0.001). Table 4 details the *F*-statistic and adjusted *R*^*2*^ for models based on both datasets, as well as the ICC for models based on *Dataset 2*. Graphs showing that the models comply with the appropriate statistical assumptions required for regression modeling can be found in Appendix A. For the MLR developed for SDNN, based on *Dataset 1*, two observations were found to have an undue level of influence in changing the slope of the regression line (as assessed with Cook’s distance). These two observations were removed for the development of this specific model, resulting in a total of 288 observations being included.

**Table 4:** The statistics for both the MLR and LLM models, which incorporate FEs and FEs + REs, respectively.

	DV	MLRs: Fes		LMMs: FEs + REs		ICC
		*F*-statistic	*R*^2^(adjusted)	*F*-statistic	*R*^2^(adjusted)	
*Dataset 1*	SDNN	39.1 (*p* < 0.001)	0.44			
	RMSSD	58.8 (*p* < 0.001)	0.55			
	SampEn	29.2 (*p* < 0.001)	0.37			
*Dataset 2*	SDNN	20.1 (*p* < 0.001)	0.33	8.6	0.65	0.48
	RMSSD	53.5 (*p* < 0.001)	0.58	75.1	0.87	0.68
	SampEn	28.3 (*p* < 0.001)	0.42	30.2	0.59	0.28

The F-statistic, *R*^2^(adjusted), and, where applicable, ICC are reported for each DV in both datasets.

*DV* dependent variable, *MLR* multiple linear regression model, *LMM* linear mixed-effects model, *FE* fixed effect, *RE* random effect, *ICC* intra-class correlation.

In both datasets, RMSSD is the HRV feature for which the variance is best explained by the IVs. It is also the DV most affected by inter-subject variability as assessed by the ratio of the variance of the random intercept to the total variance (ICC = 0.68). In the models for SDNN and SampEn, about 50% and 30% of the total variance is attributable to the variance of the random intercepts, respectively. When comparing the models with only FEs for both datasets, one noticeable difference is a higher adjusted *R*^2^ and *F*-statistic for the SDNN in *Dataset 1* compared to *Dataset 2*. The remaining statistics are comparable between datasets.

**Figure 1:**
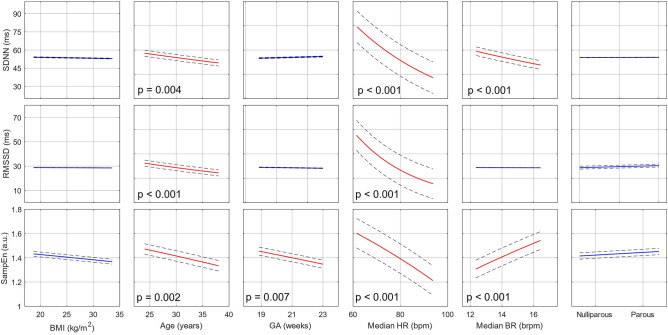
Individual regression plots showing the relationship between individual IVs (from left to right: BMI, Age, GA, Median HR, Median BR, and parity) and the three DVs (from top to bottom: SDNN, RMSSD, and SampEn) of the MLR developed for Dataset 1.

**Figure 2:**
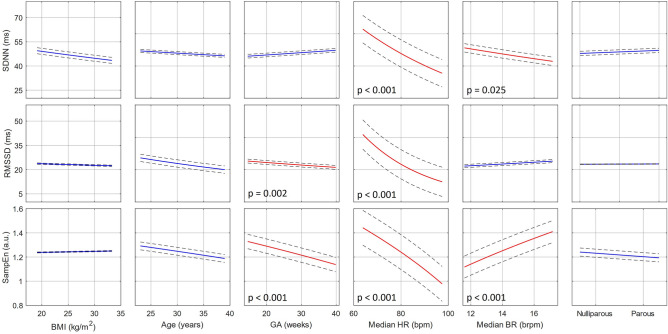
Individual regression plots showing the relationship between individual IVs (from left to right: BMI, Age, GA, Median HR, Median BR, and parity) and the three DVs (from top to bottom: SDNN, RMSSD, and SampEn) of the LMM developed for Dataset 2.

The individual effects of all IVs are characterized in Figs. 1 and 2 for *Dataset 1* and *Dataset 2*, respectively. For *Dataset 2*, the results of the LMM are plotted as this is the more appropriate model for the dataset (The results of the MLR for *Dataset 2* are visualized in Fig. S6 in Appendix B). Notice that for both datasets, SDNN and RMSSD are dominantly influenced by median HR, with a decrease in HR corresponding to increased variability. In both figures, a significant negative relationship between SDNN and the median BR can also be seen. RMSSD is also further negatively influenced by age in *Dataset 1* (Fig. 1), while Fig. 2 shows that RMSSD also decreases with advancing GA. Concerning SampEn, in both cases, this feature is influenced by a multitude of factors. Similar to SDNN and RMSSD, it is affected by the median HR, although this effect is not as dominant as for the time-domain features. SampEn is also comparably influenced by median BR. Furthermore, SampEn decreases with GA. Interestingly, this is not only seen over the long-term progression of pregnancy (*Dataset 2*, Fig. 2) but even within the 18–24-week window of *Dataset 1* (Fig. 1). Lastly, BMI and parity have no significant effect on any of the DVs in either dataset.

## Discussion

In this study, we used statistical modeling to unravel the effects of maternal demographics and cardiorespiratory factors on mHRV. Owing to the association between pregnancy complications and maternal autonomic dysfunction, there is increasing interest in the possibility of using mHRV as a screening tool for maternal health^13,14^. Therefore, it is important to establish the factors influencing mHRV in healthy pregnancies. Overall, our results suggest that we should reject the null hypothesis that median HR, median BR, age, and GA do not affect mHRV. At the same time, there is no evidence to support that BMI and parity affect mHRV.

We performed our analyses with two datasets. First, we developed an MLR using a relatively large dataset (n = 290) with a single measurement per participant to characterize the effects of our host of DVs (age, BMI, GA, parity, median HR, and median BR) on selected mHRV features (SDNN, RMSSD, and SampEn). Thereafter, based on a dataset of 29 women with a median of eight measurements taken over pregnancy, we developed an LMM to further quantify the contribution of inter-subject variability on mHRV features. To our knowledge, this is the first analysis of this nature performed in a pregnant population.

The most consequential finding is the large contribution of inter-subject variability. Not only does incorporating REs significantly improve the models for *Dataset 2* compared to models with only FEs (*p* < 0.001), but also all models have large ICC values (Table 4). For SDNN and SampEn, about half and one-third of the overall explained variation is attributable to inter-subject variability, respectively; for RMSSD, this number is over two-thirds.

Further adding to the complexity of interpreting mHRV is that it changes significantly with GA. This is evident not only from our results for RMSSD and SampEn in Fig. 2 but also from previous research reported in the literature^6,34^. These results suggest that if mHRV is used for screening purposes, the focus should be on longitudinal trends rather than absolute comparisons, with each mother serving as her own baseline. It is already possible to implement such personalized monitoring since a plethora of wearable HR monitors are available that could longitudinally and unobtrusively track trends in mHRV throughout pregnancy. Furthermore, researchers have already shown high compliance with wrist-worn monitoring of maternal HR during pregnancy^35^.

It is interesting to note the strong, negative relationship between SampEn and GA. This reduction in complexity with progressing pregnancy is seen both over the span of 16–41 weeks of gestation (*Dataset 2*, Fig. 2) as well as over the narrower range of 18–24 weeks (*Dataset 1*, Fig. 1). This downward trend in HR complexity has been previously reported as well^36^. In contrast, the effect of GA on RMSSD is less pronounced between 16 and 41 weeks of gestation (*Dataset 2*, Fig. 2) and not present over the shorter range (*Dataset 1*, Fig. 1), even though maternal parasympathetic activity is known to decrease during gestation^6,34,37^. These results suggest that complexity features such as SampEn may be more sensitive to the autonomic changes occurring within gestation than traditional time domain HRV features.

Furthermore, we also observe a significant decrease in all mHRV features with increasing age for *Dataset 1* (Fig. 1). These relationships are not evident in Fig. 2 (*Dataset 2*); however, for RMSSD the age-related effect is likely captured in the inter-subject variation. In the MLR for *Dataset 2* (Fig. S6, Appendix B), the significant relationship between RMSSD and age can be observed. For SDNN and SampEn, the lack of evident relationship could be owing to the smaller sample size in *Dataset 2* (n = 290 vs n = 29). Researchers have previously found reduced SDNN in older populations^20^. Similarly, vagal activity is also known to decrease with age. Although this reduction is typically more pronounced later in life, some studies have found a decrease within the age range of childbearing women^19,20^. SampEn, on the other hand, has been less frequently studied in relation to age. A small study found that complexity indeed decreases with age, but offers no information on the possible physiological mechanisms responsible for this change^38^. Reduced SampEn may reflect a less adaptive autonomic system in older women. While it should be noted that women in this study are within a fairly narrow age range (18–45 years), significant decreases in other HRV features have been observed between these decades of age^19,20^.

The final two demographics (BMI and parity) did not have a significant effect on mHRV features in our study. Literature on whether HRV is linked to BMI is contradictory. While the majority of studies have found a higher BMI to be associated with reduced HRV in non-pregnant participants^18,19^, others observed the opposite^39^. Parity, which refers to the number of times a woman has previously given birth to a fetus with a gestational age of 24 weeks or more, has been shown to affect hemodynamic parameters^40^. Pregnancy necessitates unique maternal cardiovascular changes. Subsequently, researchers theorize that maternal physiology may adapt more quickly in a second pregnancy, given that gestational cardiovascular needs have previously been encountered. However, we found that parity does not affect mHRV. Parity was denoted as a binary categorical variable in our analysis (i.e., nulliparous or parous). Incorporating parity as a numerical variable in the models showed similar results, though it should be noted that the number of women with a parity over one were limited. In their assessment of mHRV in the frequency domain, Al-Shadei et al. also observed no changes in mHRV in relation to BMI and parity^21^.

Finally, cardiorespiratory factors dominantly affect mHRV. SDNN and RMSSD seem to be the most strongly influenced by median HR (Figs. 1 and 2), with a higher HR associated with lower variability. SampEn is affected by both median BR and median HR. The relationship between HRV features and HR is well established in literature^16,41^. This relationship, along with the fact that baseline HR differs greatly between participants^39^ and will increase with healthily progressing pregnancy^42^, further supports the case for individualized, long-term mHRV analysis.

A major strength of our analysis is the size of the datasets used to develop the models. *Dataset 1*, which contains maternal ECG measurements for 290 women, is one of the largest maternal datasets in the obstetric literature. This dataset enabled us to establish the statistical significance of factors influencing mHRV. Moreover, to our knowledge, *Dataset 2* contains the highest number of repeated maternal ECG measurements during pregnancy that have been reported in the literature. This allowed for a unique opportunity to establish the effect of inter-subject variability on mHRV.

Still, our study has some limitations. Even by accounting for inter-subject variability, 13–41% of the variation observed in the mHRV features could not be explained. Future studies should aim to incorporate further demographics and measurements, such as blood pressure and fitness level. Lastly, owing to a lack of standardized measures of respiration in our measurements, we estimated median BR from participants’ tachograms using EMD. Although this method gives an estimation of BR which aligns with the ranges expected in a healthy pregnant population^42^, it remains an estimation. We recommend that future studies incorporate direct respiration measurements to verify our results.

In conclusion, if mHRV measurements were to be used as a screening tool for high-risk pregnancies, then age, median HR, median BR, and GA should be controlled for. Furthermore, owing to the large contribution of inter-subject variability to mHRV, assessments of mHRV should be personalized to each woman. Consequently, we would recommend the long-term tracking of trends in mHRV over periodic assessments that are compared against predefined, normative mHRV ranges.

## Supplementary Information

Supplementary Information.

## Data Availability

Data is not publicly available but may be made available at reasonable request to the corresponding author.
