# Supplementary material for: Characterizing the effect of demographics, cardiorespiratory factors, and inter-subject variation on maternal heart rate variability in pregnancy with statistical modeling: a retrospective observational analysis
Source: Sci Rep. 2022 Nov 11;12:19305. doi: 10.1038/s41598-022-21792-2 (PMC9651120; doi:10.1038/s41598-022-21792-2)
Supplement: Supplementary file 1 — Supplementary Information. [file 41598_2022_21792_MOESM1_ESM.docx]

**Appendix A**

In the following figures, we show exemplary examples of model diagnostics corresponding to the model of SDNN, based on *Dataset 1*, as well as the distribution of the REs of the model for SDNN, based on *Dataset 2.* Based on the initial diagnostics of the models for SDNN, two outlier values were identified and removed. The figures reported below are representative of the models after the removal of these outliers.

1. *Normal probability plot*


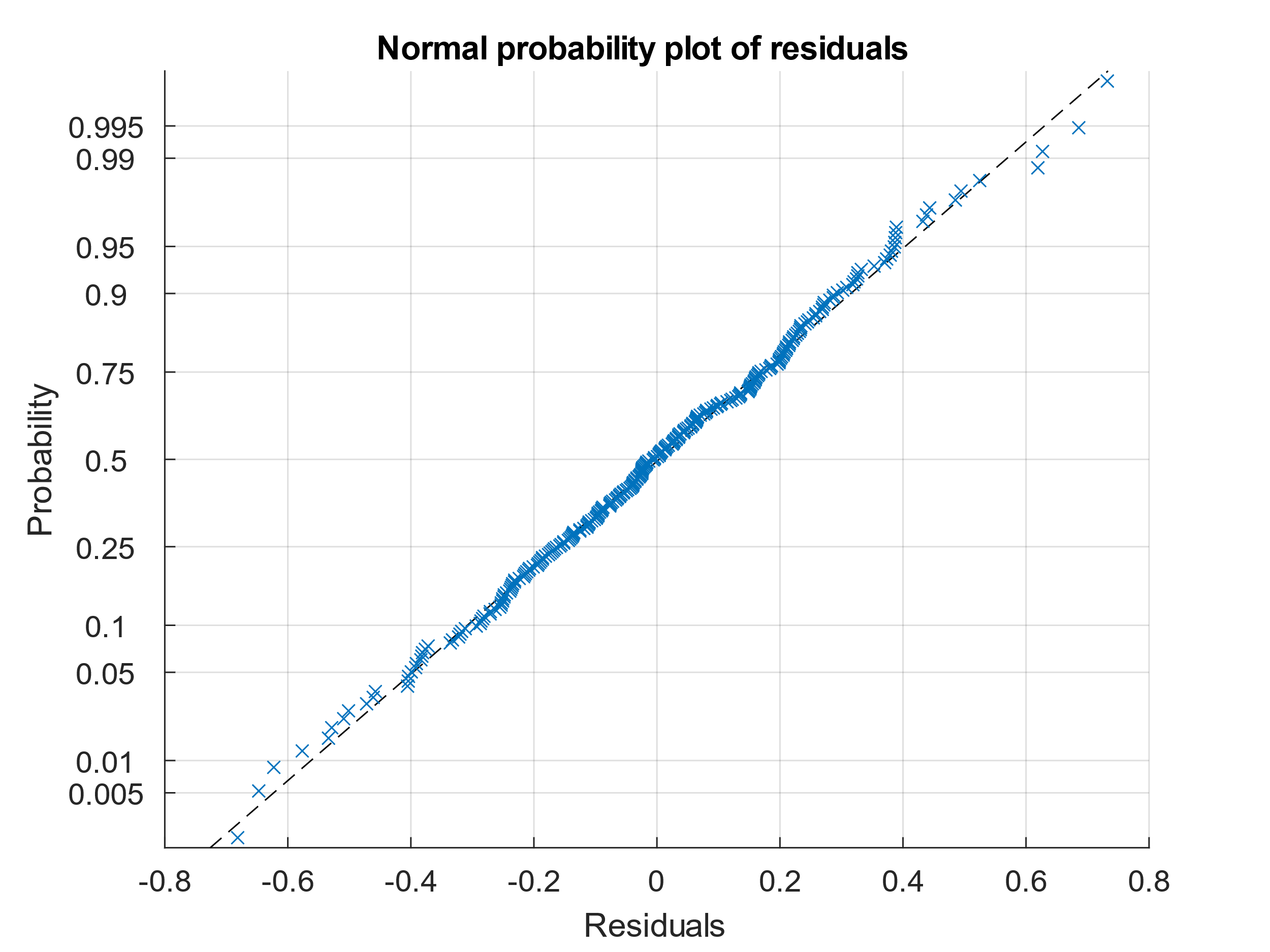


fig. S1: The normal probability plot of the residuals of the MLR model developed for SDNN, based on Dataset 1.

Other than the few outliers in the tails, the largely diagonal distribution of the residuals indicates that the distribution of residuals was overall normally distributed.

1. *The fitted values versus the residuals*


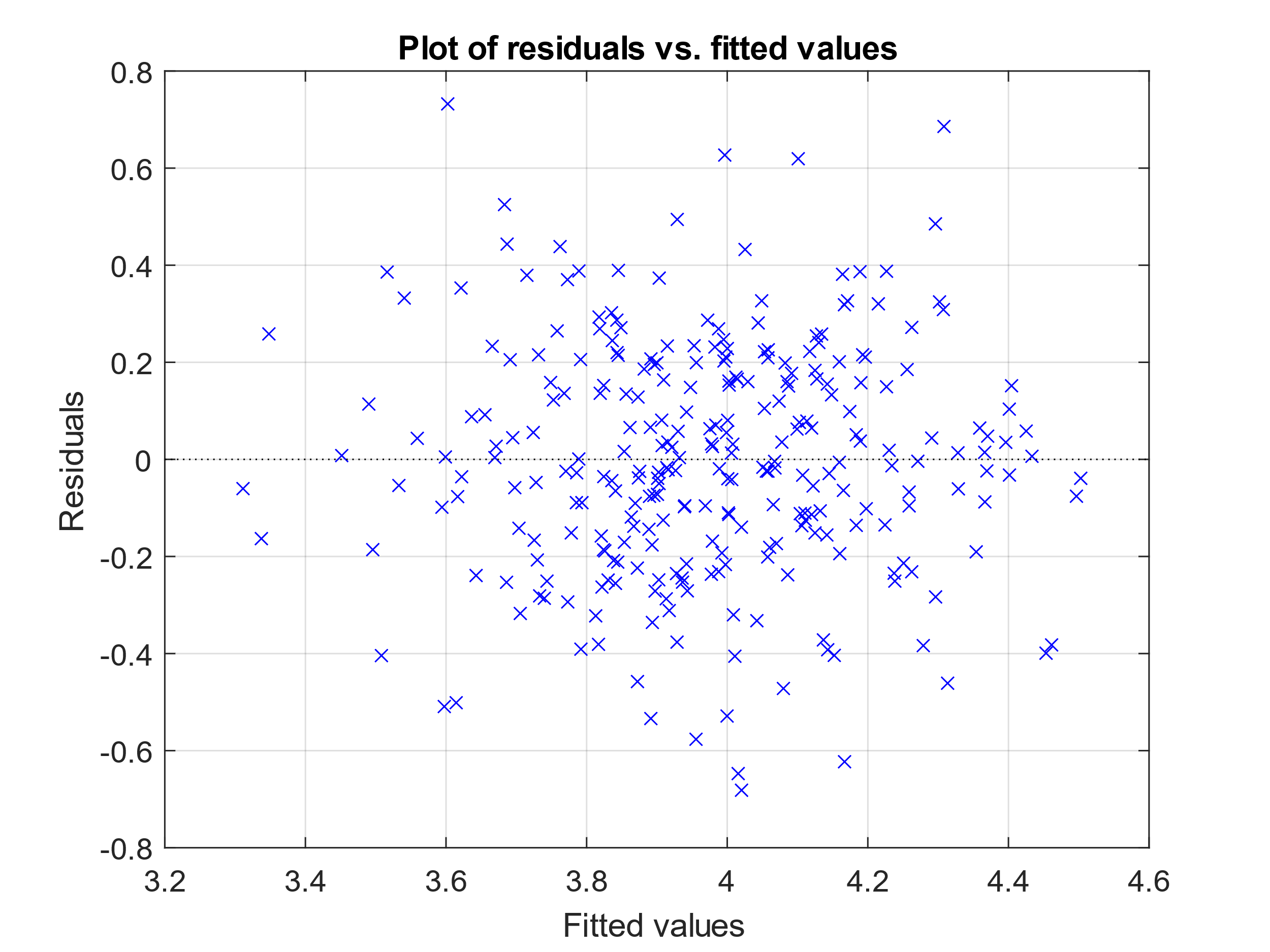


fig. S2: The residuals vs. the fitted values of the MLR model developed for SDNN, based on Dataset 1.

The residuals appear to be randomly distributed around the fitted values and have no predictive value, suggesting that the model is sufficiently homoscedastic.

1. *The plot of BMI (an IV) versus the residuals.*


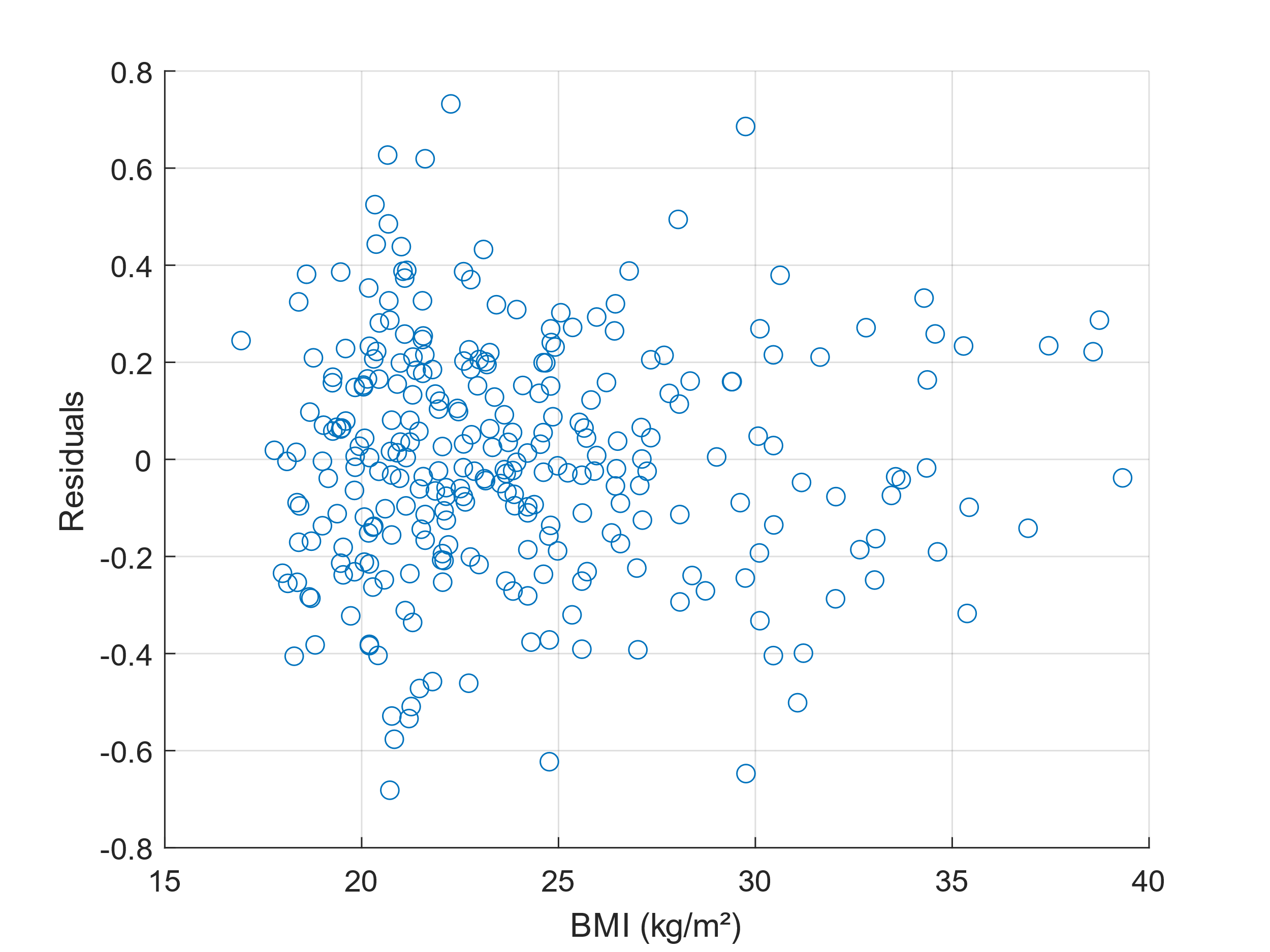


fig. S3: BMI (an IV) vs. the residuals of the MLR model developed for SDNN, based on Dataset 1.

There are no trends in the data, therefore there is no need to transform the IV prior to the model development.

1. *A plot of the residuals versus the leverage with overlaid Cook’s distance.*


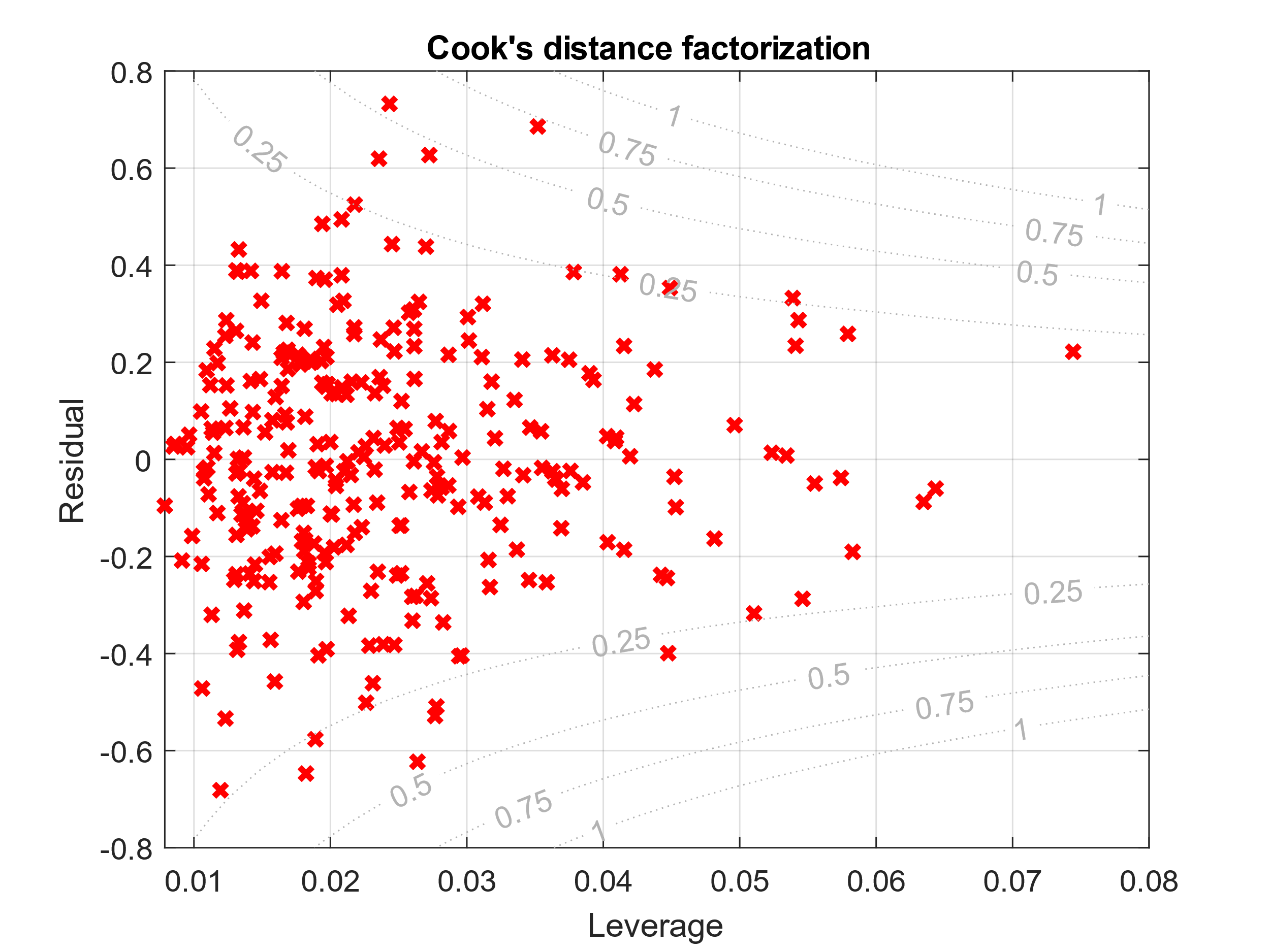


fig. S4: The leverage vs. the residuals of the MLR model developed for SDNN, based on Dataset 1, with overlaid contours of Cook’s distance.

By assessing Cook’s distance, we see that all observations fall within a Cook’s distance of less than one (which serves as a rule of thumb). Furthermore, all observations have relatively low leverage values. Subsequently, there are no notable outliers.

1. *Histogram of the REs*


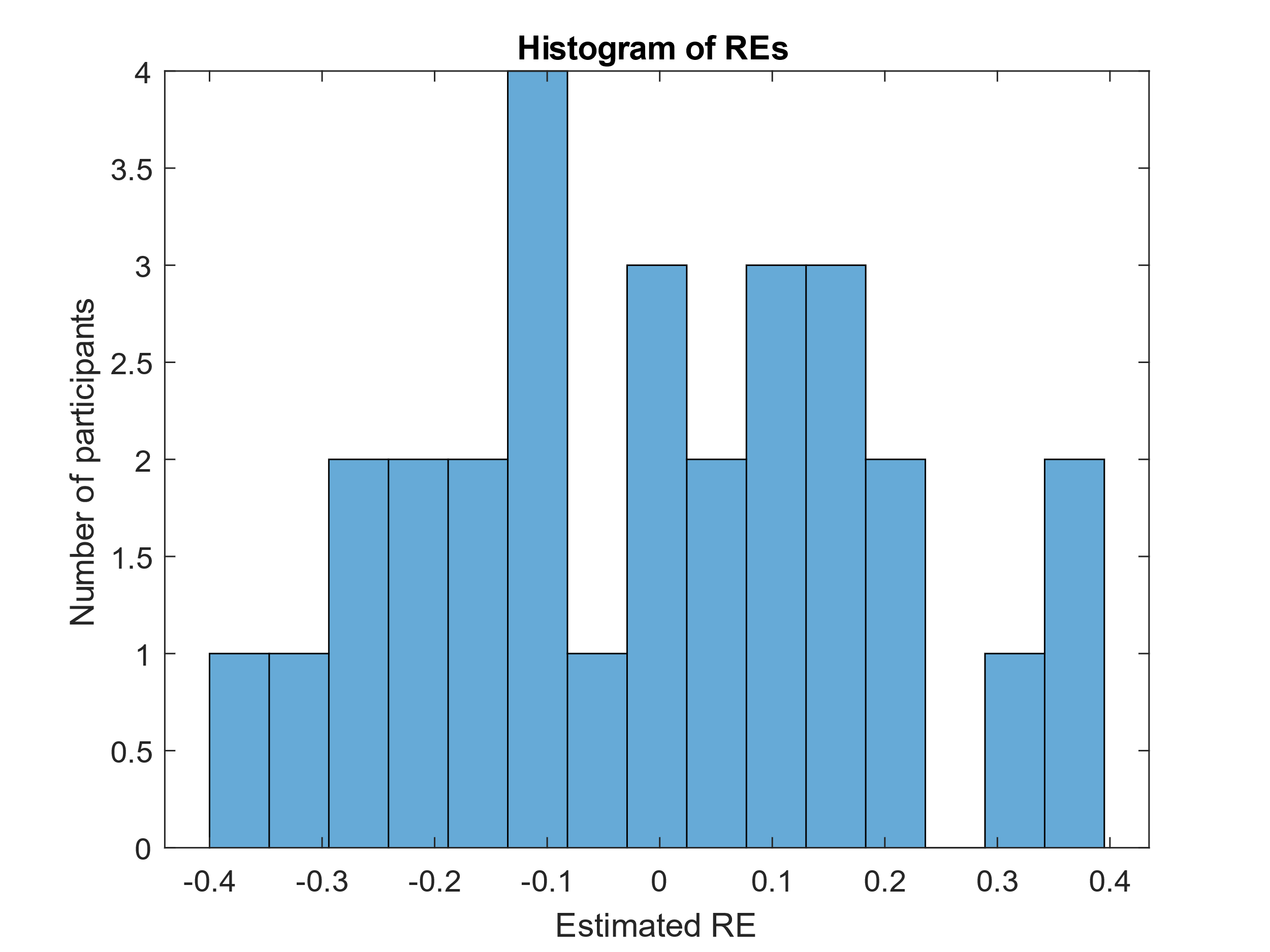


fig. S5: The distribution of the REs for the LMM model developed for SDNN, based on Dataset 2.

The REs appear to be approximately normally distributed.

**Appendix B**


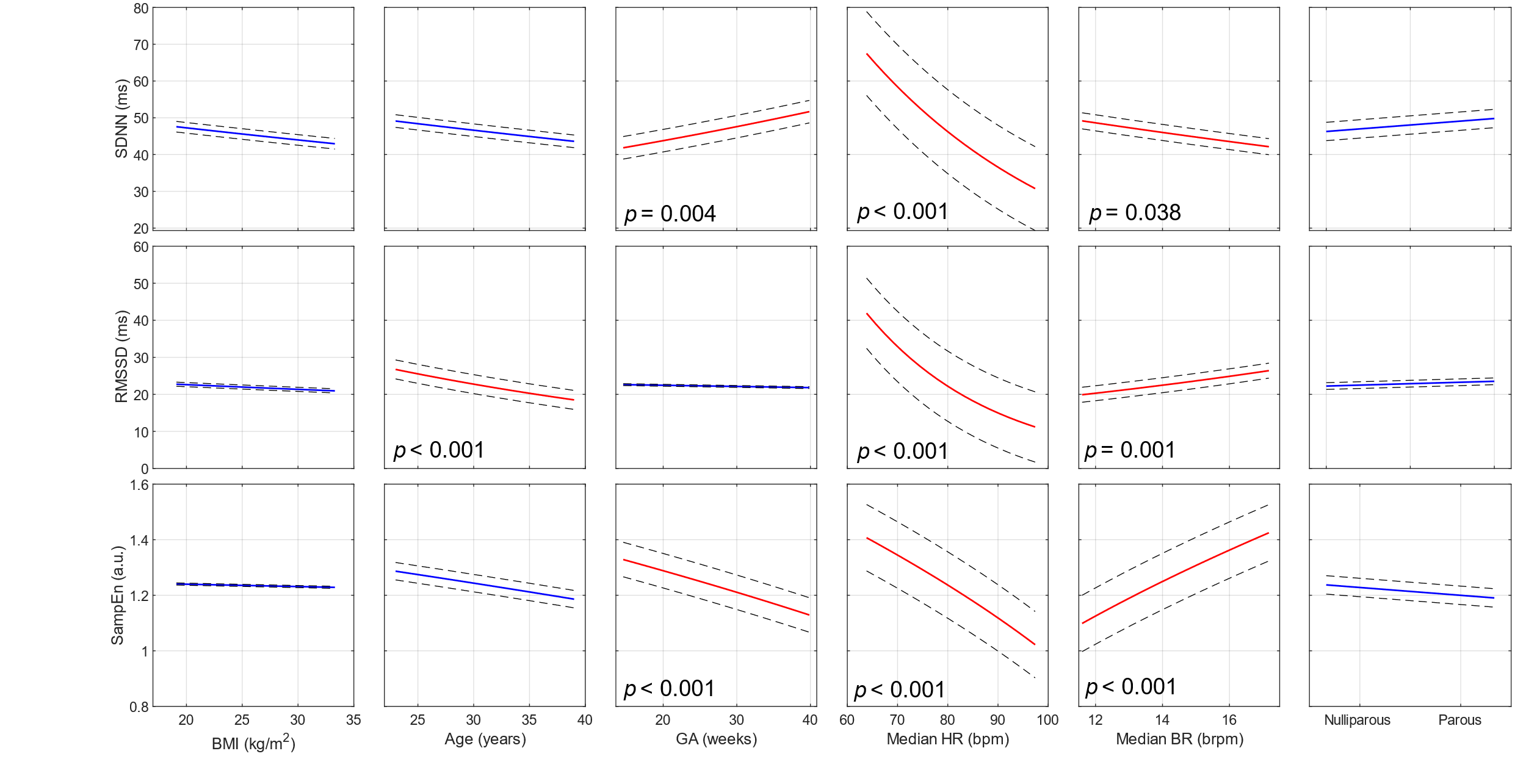


fig. S6: Individual regression plots showing the relationship between individual IVs (from left to right: BMI, Age, GA, Median HR, Median BR, and parity) and the three DVs (from top to bottom: SDNN, RMSSD, and SampEn) of the MLR for Dataset 2.
